# General Therapeutics

**Published:** 1837

**Authors:** 


### REVIEWS

AND

### BIBLIOGRAPHICAL NOTICES.

---

### Art. IV.—General Therapeutics.

*General Therapeutics, or Principles of Medical Practice; with tables of the chief remedial agents, and their preparations, and of the different poisons and their antidotes.* By Robert Dunglison, M. D., etc. etc. etc. Philadelphia, Carey, Lea & Blanchard, 1836.

It is usual to commence the review of an elaborate work on science, by a declaration of the importance of the subject treated of, and the high claims preferred by it upon our consideration. This mode of bespeaking the reader's interest, in the matters to be discussed, may prove effectual in eliciting the reviewer's effort to make the analysis of the book in hand acceptable, by a fair and lucid exhibition of its essential features, and thus the great end of this method of promulgating truth through the periodical press will be attained. We sit down to the review of the above named work with an anxious wish to do the author justice. But we feel a still stronger desire to take a just and thorough view of the great questions involved in the discussion to be submitted in this article. We feel well assured that the subject of therapeutics is one of very great importance to the cause of practical medicine. And we are constrained to say, that the distinguished author of the work has not done ample justice to the wide theme.

After a careful perusal of the work, we are compelled to give it as our conviction, that Professor Dunglison's high reputation will not be advanced by the publication. In a work of five hundred and seventy-nine pages, we, of course, expected some things of doubtful utility, but our minds were astounded to witness the strong evidences, which are so abundantly diffused over its pages, of the hasty getting up of the work.—There are many, very many, excellent things to be found in these five hundred and seventy-nine pages—we sincerely wish that they were still more copiously distributed through the entire body of the composition.

The author commences his first chapter by defining therapeutics, which he tells us, "comprises the doctrine of the management of disease."

Philosophically speaking, the science of therapeutics should, we think, be limited to a just comprehension of the true curative indications to be fulfilled in disease, and the appropriate methods for the fulfilment of such indications. It differs from materia medica, in this very prominent and important point—that it is the guide—the true director of a scientific administration of drugs. Materia medica comprises the remedial resources by which disease is combated. Therapeutics stands between pathology and materia medica, deriving its light and authority from an enlightened conception of disease, its nature, seat, degree of activity, and stage, it points out to us the ends to be attained by medical interference for the restoration of the patient to health, which ends of remedial agency, materia medica furnishes appropriate means to accomplish.

Professor Dunglison, we think wrong in the remark made in the second sentence of his first chapter, that, "generally the term (therapeutics) is restricted to a description of the *modus operandi* of medicines," this declaration seems in contradiction to the meaning of the next passage. Under the division which obtains in our medical institutions, "Therapeutics is made to embrace the general principles of medical administration, and the indications which the different articles of the Materia Medica are capable of fulfilling." We know not a more barren and intangible species of medical reasoning, than that employed by writers on Materia Medica in their explanations of the *modus operandi* of medicines.

Now, our author, although he rebukes in the following terms, the injudicious manner in which writers on Materia Medica have treated the questions connected with that department of the science of medicine, yet he is not free himself from a serious fault in his mode of discussing the principles of a just and enlightened therapeutics. "The object of this work is to attempt to lay down the great general principles of therapeutics, and to avoid detail. Of the evils of too great detail, we have indeed, the most marked examples in some of the existing works on therapeutics and Materia Medica, and it is utterly impossible for the student to rise from their perusal, with other feelings than those of confusion—inextricable confusion." page 457.

We can, most conscientiously, reiterate the remark just quoted, and make a most unforced application of it to the work under review. Instead of simplifying the subject, we think he has, in a most marvellous degree, made confusion still more confused. To exemplify our stricture, let us begin with the beginning, and with *coup d'œil* pass through the work.

"There is no branch of medicine with which the therapeutist ought not to be acquainted. To be a good therapeutist, requires not only that the individual shall have had extensive opportunities for witnessing disease, but also that he shall have read, extensively, the recorded observations of others. It demands, too, the utmost powers of discrimination; hence, the varied knowledge which the physician ought to possess, and the learning and dignity of the science." All this is very well; against such a representation of the superior claims of therapeutical knowledge, we demur not. But now commences, according to our judgment, the objectionable parts of the work. Several pages are devoted to a common place discussion of the medicating powers of nature, and in the form of a note there is a quotation from an English poet, to show what the medical superstition of his day was, in reference to the restorative efficacy of dressings, applied, not to the wound, but to the sword which inflicted it. This is not altogether in place in such a work, though it may have occupied an appropriate position in a history of Materia Medica. After devoting several pages to this thread-bare topic of the *vis medicatrix naturæ*, we are gravely assured by our author, that "the existence of such an instinctive power, can neither be denied nor lost sight of in the treatment of disease. The error has been, that undue weight has been attached to it, so that the practitioner was altogether guided by its manifestations, in laying down his indications of cure; and if no such manifestations appeared, he waited vainly—and too often most unfortunately—until the time had perhaps gone by for the administration of efficacious agents. So this system of waiting, or expecting the term *medicina expectans—la medicine expectante*—was appropriated. The followers of Stahl—the great apostle of this doctrine—supposed a power to exist in the system, for expelling morbific causes, and of re-establishing equilibrium when disturbed." But after all, the author candidly admits that "there are but few cases in which this power can be trusted to." Still there is this consolation that, "although we may discard the notion of efforts of nature, there is no doubt that good occasionally results from spontaneous discharges."

Why then this waste of argument to prove the medicating powers of nature, when they cannot be *invoked*, to use a very favorite word of the author, in the management of disease. The reader is referred to some suggestions on this point, in the last number of this journal, contained in a lecture on the indications of cure in disease. We next have several pages of very judicious remarks on theory, facts and experience—which we think deserving of careful consideration.

At page 19, we have the following reflections. "It is by empirical trials, that we become informed of the properties of any medicinal agent, after which sound physiological and pathological knowledge must suggest its correct application."

"The great object of the science of medicine is to remove disease. Hence the pre-eminent importance of therapeutics." "It is the therapeutical *indications*, that vary so much with medical theories. By indication, is meant, the end to be had in view in the administration of remedies." "The fundamental object in every indication, is to put a stop to, or mitigate the disorder in the organic actions, and to remove any alteration that may have supervened in the tissues, consequent on such disorder." "The nature of the disease, or the precise species of vital modification of tissue, that gives occasion to the morbid phenomena, must be the base of every therapeutical indication." Again, the author avers that "to know the organ, and the disease under which it is suffering, is more than half the cure; the selection of medicinal agents, and their mode of action then become beautifully and systematically clear. Experience—true observation—and sound reasoning, join in the elucidation of the subject, and success—as much as the case will admit of—cannot fail to follow the exertions of the physician."

In the correctness of some of the above positions, we confess that our minds are sceptical. The appropriateness and efficacy of any plan of treatment are to be ascertained by a well weighed and thoroughly tried series of clinical applications of remedies. The author concedes this—but at the same time declares that "the nature of the disease, or the precise species of vital modification of tissue, that gives occasion to the morbid phenomena, must be the base of every therapeutical indication." Does he mean to inculcate the sentiment that, before we can scientifically and successfully prescribe in a case of disease, that we must first ascertain the essence, or peculiar nature, of the morbid train of processes going on in the organism? If so, then must the practitioner stand in terrible suspense of mind at the bedside of the sick. For who yet understands "the precise species of vital modification of tissue" that obtains in inflammation, or in fever, or in irritation? Have we yet settled down on any positive views of the nature of inflammation—whether it consists in increased or diminished action—or of fever, whether it is a truly idiopathic complaint, or only symptomatic of lesion of some vital organ—and if fever is but a symptomatic complaint, is the *fons mali*, the brain, or stomach, or spinal marrow, or liver?

The author tells us that we are not to prescribe for symptoms; but for what then are we to prescribe? If he had directed us not to prescribe for one symptom to the neglect of the others, we should have concurred in the statement. But how do we recognize disease but by the symptoms? If a patient has a full, strong pulse, with intense head-ache, hot skin, foul tongue, etc., do we not prescribe for the symptoms when we bleed him? Where, in such a case, is the essence, or "precise species of vital modification of tissue, or of the organism? do we know any thing about it? Our curative indication is taken from the symptoms, and not from the essential character, or peculiar modification of vitality in the case, except so far as that is made known by the symptoms. And our confidence in depletion in the case of disease just adduced, arises not from any knowledge we possess of the peculiar essence of morbid action, but from an experience, personal or derived, or both, acquired by our own trials, and the reported experience of others, of the efficacy of certain plans of treatment, in certain states of the system.

The value of morbid anatomy is limited to diagnosis and prognosis—to an identification or detection of disease, and the seats it occupies. The therapeutics of the case spring from a different quarter, and is to be perfected by other methods than those of morbid anatomy, or transcendental pathology. We call that transcendental pathology, in this connection, which pretends to a perspicacity of vision, beyond the sober bounds of a just experience. Let us illustrate our meaning. Suppose we are called to treat a case of fever obeying a paroxysmal rule—that is, it recurs regularly every day at 12 o'clock M.; it is ushered in by a chill, fever ensues, and terminates by a copious perspiration. It may be a case of fever of miasmatic origin; that is, a regular intermittent; or it may prove a case of hectic fever, from tubercles in the lungs. These distinct kinds of paroxysmal seizure, we have seen mistaken the one for the other. We have known hectic fever arising from diseased lungs, treated with wine and quinine, with what effects, the enlightened reader need not be told. Now, the value of morbid anatomy consists in this highly useful practical result—that upon opening two bodies, one of which has died of intermittent, and the other of genuine hectic, we are informed of the true character of each disease. That one was primordially a local affection of a vital organ, going off fatally, in a descending train of febrile excitements, emaciation and debility—and that the other was an idiopathic disease, which arose from a febrific agent acting on the system in a manner, not satisfactorily made out to all parties. The symptoms were, in some respects alike, and yet, when carefully scrutinized, they were found to vary in some essential points. But the value of a particular kind of treatment for each, respectively, is declared to us by experience alone. Pathological anatomy tells us that there is a veritable difference in the seat of these two, in some aspects, analogous cases; symptomatology assures us that there is a pointed separation in the two sets of phenomena witnessed in these two instances of disease, and experience has taught us that intermittent is to be cured by bark, the great antiperiodic remedy, and that hectic is to be mitigated, but not cured in the instance brought forward, by a soothing plan of treatment.

We entirely coincide with Professor Dunglison in the opinion advanced by him—that the therapautics founded on the humoral pathology have passed away, though it is manifest, from the pages of the work under review, that the hypothesis is still fondly cherished by our author.

We were much disappointed in not finding in this system of therapeutics, some well generalized, comprehensive, and available curative indications given. And the confused array, the medley host of remedies crowded together under different heads, such as are found under the author's division general, and divisions subordinate, indicate any thing but a philosopical exposition of the important subject of therapeutics.

The following is our author's classification of therapeutical agents:

| | | | |
|---|---|---|---|
| I. Vital agents. | Excitants. | Increasing action generally, or locally, or both. | Excitants proper, Tonics, Anthelmintics, Astringents, Emetics, Cathartics, Emmenagogues, Abortives, Diaphoretics, Errhines, Sialagogues, Diuretics, Expectorants, Sorbefaciants, Revellants, Antispasmodics, |
| | | diminishing action, directly, or indirectly. | Sedatives proper, Narcotics, Refrigerants, Nauseants, |

II. Chemical Agents.
- Antacids,
- Antalkalies,
- Antillithics,
- Disinfectants.

III. Mechanical Agents.
- Demulients,
- Diluents.

We conceive that Begin's arrangement is much more philosophical and just.

Begin's General View of Therapeutical Agents:

*Therapeutical actions.* They all take place, first, by the immediate action of pharmacological substances on the living tissues; 2d, by the continuity of the parts; 3d, by the contiguity of the organs; 4th, by sympathy;—5th, by abf sorption of medicinal particles.

- Debilitations, They may be
  - 1st. Limited to the surfaces in which the remedy operates, which are
    - The integuments,
    - The organs of the senses,
    - The genito-urinary organs,
    - The organs of respiration and voice,
    - The digestive viscera,
    - The serous membranes,
    - All parts accidentaly denuded.
  - 2d. Extended to one of those 3 great systems.
    - The blood,
    - The nerves,
    - The lymphatics.
  - 3d. General, or involving every part of the living economy.
- Direct stimulations.
  - They are susceptible of the same divisions as debilitating medications.
- Revulsive, or indirect medications
  - They are also local, and may be applied to
    - The skin,
    - The secretory organs,
    - The cellular substance,
    - The organs of locomotion,
    - The digestive viscera,
    - The brain,
    - All parts denuded by solutions of continuity.

Agreeably to Begin's classification of remedial agents, we have three great leading therapeutical indications to guide our clinical application of these substances. The first is, to debilitate; the second, to stimulate; the third, to produce revulsion. According to Prof. Dunglison, our leading indications will be to act, first, vitally; second, chemically; third, mechanically.—And then his subordinate indications under the vital agents, would be, first, to excite action generally, or locally, or both; second, to diminish action directly or indirectly. Let us now see what singular positions some of the most effective articles of the materia medica are made to assume in our author's collocation. Calomel and blue pill we have placed with sulphuric ether, ammonia, capsicum, etc., under the head of excitants proper, and in looking under the table of cathartics, we have these mercurials merely put down, and reference made opposite to the table of excitants.

Under the general division of vital agents we have excitants, and under that division we have stimulants, (excitants proper) antispasmodics and tonics, placed under the same category with emetics, cathartics and diaphoretics. Tartar emetic and cinchona, alcohol and epsom salts, ipecacuanha and musk, collocated under the same general division of vital agents which act by increasing action generally, or locally, or both. Narcotics and nauseants diminish action, directly or indirectly.—Tartar emetic and ipecacuanha increase action under the head of excitants, whilst they are made to pass through a grand act of proteism, under the form of nauseants, and as such, diminish action.

We are astonished that our author should have retained abortives, errhines, sialogogues, sorbefaciants, refrigerants, antacids, anthalkalies, disinfectants, demulcents and diluents, when it was so easy, under his other heads, to have distributed the few, and generally inert articles, which he has enumerated under the above respective divisions. It affords a sad evidence of the confusion and the defect of simplicity, in the arrangement of the articles of the materia medica, which still prevail, to see such a medley of obsolete (as we had fondly imagined) terms thrust in to make up a regimental row, or rather militia mob, of pharmacological materials.

We did suppose that the march of science was characterized by a more free and disencumbered step—that the weights hung upon her by a vain display of drapery would be removed—and that henceforth we were to witness a far more dignified simplicity of costume than had ever clothed her august body.

But Professor Dunglison has thrown around one important department of one very important branch of science, a meretricious array of ill-assorted phraseology, and instead of leaving the subject of therapeutics more attractive, has rendered it more repulsive—for instead of elucidating and simplifying it, he has obscured many parts, and rendered the whole subject more difficult of comprehension.

Therapeutics should deal mainly and principally in the great leading indications of cure in disease; and the articles of materia medica should stand in an ancillary attitude in relation to such indications. A wide, and at the same time correct indication of the common divisions of mateira medica should be made. The generalizations springing out of such an induction, should harmonize with the different exigencies of morbid action, which we are called on to treat. Thus we have, according to the arrangement of Begin, debilitating medications, to meet those conditions of the system, often occurring in disease, in which it is imperative upon us to bleed, &c.; and we have stimulating medications, which are called for in other states of the organism—and revulsive medications to produce counter-action and counter-irritation.

We conceive that Begin is too much a visionary in matters of clinical experience—that he is too deeply and injuriously embued and pervaded by the over-refinements of the French School of Medicine.

A more extended and useful scheme of therapeutical indications should be comprehensive enough to embrace all our valuable means of treatment, and should meet and correspond to the different morbid processes which obtain in the disordered movements of life.

Thus we should have arranged under the great, and often governing indication of reducing action, blood-letting, emetics, cathartics, nauseants, etc., to meet those inflammatory states of the system which often arise.

Next, we should, under the head of the indication to restore the secretions, place those articles which have the most efficient agency in carrying off abnormal action through the emunctorial outlets.

Then we have tonics to meet debility, and stimulants to excite in a more transient manner—and derivatives to derive action by counter-irritation, and alterants to modify, or alter action; and narcotics to subdue nervous irritability.

As to the disputed point of the modus operandi of medicines, what has that to do, legitimately, with therapeutics. This, we are aware, is not the prevalent conception entertained on this point. But after all the interminable war of words, which has been waged by the belligerents, among the humoralists, chemists, mechanicians, mathematicians, solidists, and vitalists, where stand the great body of judicious physicians in reference to this litigated question? Why, they stand, whilst engaged in their warfare with disease, at the bed side, upon the solid grounds of correct pathology and therapeutics. Do they enter into any problematical, debateable matters concerning the *modus operandi* of medicines? Not if they possess sound, discriminating, practical minds, which entitle them to the appellation of eclectic practitioners.

They take as ultimate facts, that tartar emetic nauseates and vomits; and that calomel acts on the hepatic apparatus, and salivates, without pausing to solve the problem whether tartar emetic is absorbed before it acts, or whether calomel medicates the blood or not.

Our author says that "the modes in which the agency of remedies is exerted, are chiefly as follows: 1. By *simple, direct, or local action.*" "2. By *indirect or general action.* This is the mode we commonly invoke, in the administration of remedies." "The manner in which this indirect effect of medicines is induced, is as follows:

*a.* Through the nerves." This the author enlarges upon in a very satisfactory manner.

"*b.* Through absorption." The author tries to establish this position, but not in a satisfactory manner, agreeably to our judgment. He tells us that "the method of administration by friction, is called the *Iatraleptic*, (from *iatros*, physician, and

*aleipho*, I anoint:) and one which consists in placing our remedies in contact with an abraded or vesicated surface, the *endermic*, (from *en*, in, and *dermis*, the skin.)"

"*c. Through revulsion.*"

By an endeavor to erect our indications of cure on the author's *modus operandi* of medicines, we shall find much imprecision and incertitude to spring. The general doctrine that remedies act through the nerves, by local and general agencies, leaves the matter in a state of the greatest latitudinarianism. Can we establish any indication upon such a reference of medicinal operation? And then there rests a similar cloud of ambiguity upon the modus agendi of medicines, through absorption. Who ever prescribes a remedy with such an indication governing the prescription? Who now talks of medicating the blood?

There is more precision and truth in the last mode, that by revulsion, for on that we can found a curative indication, and often avail ourselves of this method of procuring restorative effects from the administration of our therapeutical agents.

The author adopts, with but slight alteration, Dr. Thompson's classification, and he makes heavy draughts on his pages, in his discussion of the circumstances which modify the indications of cure in disease. These circumstances are "age, sex, original conformation, habit, climate, mental affections, professions, and way of life, as well as the causes, period, and seat of the disease."

Professor Thompson enumerates seven of these modifying circumstances, in his consideration of the action of medicines.

Begin speaks with some particularity of the following "circumstances which contribute to modify indications in the treatment of diseases."

"These circumstances relate, on the one hand, to the age, temperament, sex, profession, habits, strength or debility of the patient; on the other, to the causes, the seats, the intensity, and the periods of diseases."

Here we see our author and Begin laying down the above named circumstances of age, &c. as modifiers of the indications of cure, while Professor Thompson lays them down as modifiers of the action of remedies.

There seems an apparent incongruity and oppugnancy between these respective modes of contemplating a plain practical concern in our science. But upon a little reflection, all such seeming incongruity and oppugnancy will vanish. Any circumstance which modifies the operation of remedies, should modify, in a corresponding degree, our therapeutical indication. Say, for an illustration of our meaning, that we are aware that our patient has been in the habit of taking opium for a neuralgic complaint, and being aware also, that where intense pain exists, we may, without any apprehension of undue narcotism, administer enormous doses of this anodyne; with these two modifying circumstances present, our indications will be accordingly modified. The action of the remedy constitutes an essential portion of the integral ground-work of the indication. We know that pain exists—but suppose we knew of no remedy to relieve it, what indication of a precise practical nature could arise? But being assured that pain of a certain character, depending on, or connected with certain appreciable states of the organism, is controllable by remedies termed anodynes, the proper indication of treatment rises from disease, and a familiar means of administering relief. And thus the leading indication of restoring the secretions is often made available to high and diversified practical purposes.

In fever we often procure a solution of the disordered state of the animal economy by giving those remedies which act in producing a restoration or augmentation of the secretions of the liver, skin, kidneys, etc.

"Under different states or conditions of the body, says our author, remedies are found to produce the most various effects.

During the existence of spasm in any portion of the system, opium may be given in immense quantities, without inducing its wonted action. I have sat by the bed-side of a delicate female, laboring under the *cholelithus means* of good—that is, under gallstone, in its progress along the ductus communis choledochus—to whom I have given the tincture of opium by the tea-spoonful, until she has taken upwards of an ounce, yet without any stupor following its administration. In like manner, in neuralgia, extreme doses of narcotics may be demanded, as well as in mania and melancholia—diseases in which the great nervous centre can be impressed with extreme difficulty.

It is but lately, that an interesting lady, laboring under puerperal mania, took twelve grains of solid opium in the period of twenty-four hours; and an engineer of one of the Philadelphia steamboats, affected with severe neuralgic attack in the intestines, took fifteen grains in the same time, without the least evidence of narcosis." Under our Hospital Reports, p. 64, reference is made to a case of delirium tremens, in which we successfully employed a still larger quantity than that stated by our author. And we have been told by our friend, Professor Rives, that in the case of a young lady, in this city, afflicted with neuralgia, he has known as much as sixty-four grains of acetate of morphine to be given within the space of three hours, and the effect was, to procure a removal of the pain, with no extraordinary degree of narcotism. At another time, within three hours, she took three hundred and eighty grains of solid opium, with only similar results to those which followed the sixty-four grains of morphine. Her habitual use of the narcotic, and the intense pain she experienced, thus remarkably modified the action of this drug.

"It is astonishing," says our author, "to what an extent narcotics may be borne with impunity, where a habit of resistance has been acquired, by long protracted indulgence. Dr. Russel, in his 'History of Aleppo' states, that a Turk of the name of Mustapha Shatar, an opium eater in Smyrna, took daily, three drachms, or 180 grains of opium; and in the 'confessions of an English opium eater,' the author is affirmed to have consumed, at one time, eight thousand drops of laudanum, daily. If we consider that 25 drops of laudanum are equal to one grain of opium, this would make 320 grains, or upwards of five drachms in the day."

At page 19, our author thus writes—

"Indications of treatment have necessarily been influenced by the dominant medical sect. The humorist, or humoral pathologist, who looked to the fluids as the cause of all maladies, directed his attention to the removal of a fancied acridity, acidity, or alkalescency of the humors; or to evacuate them after they had experienced a kind of maturation or preparation, which he called *concoction*. The mechanical philosopher attended to the permeability, or the contrary of the vessels; the effect of gravity and the like; and his indications were based upon his ideas on these matters. But these systems, and the therapeutics founded on them, have passed away; not, however, without having left useful mementos of their existence; for it is obvious, that the conditions which they invoked cannot be wholly disregarded: the evil with those pathologists was, that they assigned to them too prominent a rank in the causation of disease, and that they attended to them to the exclusion of more important agencies."

Under the head of Chemical Agents, the author assures us, that "of late years, a much greater degree of attention has been paid to morbid conditions of the blood, and to the effects of therapeutical agents upon it; and now, that the horror of the humoral pathology has abated, and indeed almost vanished, fresh investigations will be made into its varying condition in disease, and into the best methods of restoring it to the healthy state." p. 482. Speaking of antalkalies, he gravely tells us "that sugar is capable of modifying, considerably, the condition of the circulating fluid, is sufficiently exhibited by the observation, first made, perhaps, by Professor Hegewisch, of Keil, that a solution of sugar produces the same alteration in the color of black blood as the saline solutions. It changes it to a bright arterial hue." p. 496. And again, at p. 502, the author says, that acids "may not only pass into the blood, and act chemically on that fluid, but invigorate the digestive apparatus, and prevent fresh deposition."

Now let the intelligent reader pause, and answer this query —does this language, coming from so distinguished an author as Professor Dunglison, evince that "these systems" (the humoral pathology and the mechanical philosophy referred to by our author) "and the therapeutics founded on them have passed away." And let the enlightened therapeutist decide this great question of practical medicine—whether these fancied states of the blood, acid, alkaline, and carbonaceous, are ever made the foundation on which he establishes an indication of cure. Sugar is a pleasant medicine for children, and may act most efficiently as an alterant to a bad temper, but if it medicates the blood in any way, we demand better proof than that adduced by the author.

From the suggestions of the author on the value of chemical agents, and their capabilities in acidifying, dulcifying, renovating and purifying the blood, and of impregnating it with antidotes, antacids, antalkalies, and antiseptics, we may derive a strong hope that the blessed light of humoralism will, with unclouded irradiation, once more rise upon the science of medicine, and that our books of practice will be full of concoctions, and despumations, effervescences and ebullitions.—Happy era, we hail thy coming dawn! Therapeutical science once more will plant its battery of medicinal war upon these flights of fancy, and disease will be as easily cured as it will be comprehended.

In sober earnestness, we deplore that such loose, intangible, and incomprehensible pathology, with such puerile therapeutics, should issue from such an eminent quarter. Sugar to change "the condition of the circulating fluid," acids to "act chemically" on the blood! Sweeteners and acidifyers! But we are done. We pass to a more pleasing part of our task. Freely have we criticised—perhaps, too boldly have we pointed out what we conceived errors—glaring errors, in the work before us. We shall now dwell upon those parts of it which deserve, as we conceive, our approbation.

We, in the first place, consider the learned author as deserving of high praise for his zealous labors in behalf of the science of medicine. There is not, in the United States, a more assiduous cultivator of our science, than our distinguished author. The fruits of his varied efforts are in the hands of thousands of the profession. The name and reputation of Dunglison is sculptured in abiding characters on the temple of American medicine. Besides the present work, the most imperfect of his different productions, and evidently thrown off in too great a hurry, Professor Dunglison has given the profession within a few years, an erudite "New Dictionary of Medical Science and Literature," an excellent treatise on the "Elements of Hygiene," also an extensive work on "Human Physiology." His "Medical Library and Intelligencer" is deserving of honorable notice, and will sustain the well merited reputation of the editor.

We are glad that Professor Dunglison has directed his attention to the subject of therapeutics, and that he has made an attempt to elevate this essential branch of our science into a more commanding position, and to confer upon it additional attractiveness. The arbitrary and unscientific manner in which the names of diseases have been put in juxtaposition with the remedies, which writers on materia medica assure us, are proper to be brought in requisition for their cure, has tended to make the whole subject one of empirical prescription, and of routine authority. Of what avail is it for the student to be told that venesection is useful in inflammation, unless informed what great governing indication is met and fulfilled by the lancet. Inflammation is a generic term, denotive of a variety of vascular lesions; but neither the symptoms indicating the presence and seat of this morbid action, nor the appropriate methodus medendi in its details, are brought before the mind, in our works on materia medica. These topics belong to the theory and practice of medicine.

Yet some reference should be made to the particular states, or varieties of inflammation, and the indications of cure should be deduced from them, in order to a scientific treatment of any given case. Take an example. A patient is suffering under pneumonitis—this is recognised by well known symptoms, such as pain in the chest, difficult respiration, cough, full, hard pulse, and hot skin. What is the indication of cure in such a case? Simply the rapid reduction of vascular activity, by blood-letting. This is the first step in the methodus curandi. But suppose there is connected with the case a depressed state of the general circulation—and that we have announced to us by the stethoscope the commencement of red hepatization. What now is the indication of cure? General blood-letting—nay, even a local abstraction of blood by leeches or cups applied to the chest, is inadmissible, from the subdued condition of the vital energies—and even if admissible, would, perhaps, prove unavailing in arresting the rapid organic lesion, set up in the pulmonary texture. Counter-irritation may prove beneficial—but our main reliance is to be placed in alterants, such as tartar emetic and mercury. American practitioners of medicine, place, with just grounds of assurance, their greatest amount of confidence in the alterant agency of mercurialism.

But would it be scientific, and in accordance with the legitimate authority of an enlightened therapeutics, for a writer on materia medica to assert that blood-letting was good for pneumonitis, and that tartar emetic and calomel were likewise good, without adverting distinctly and pointedly to the variant states of the system in which each were respectively good, and without impressing on our minds, the peculiar character of the therapeutical indication to be fulfilled?

The theory, or doctrine of indications, stands as a line of light between the pathology of a case and those articles, or measures of materia medica, which can be brought successfully to bear upon the kind and degree of morbid process going on in the system.

Writers on materia medica have unhappily multiplied these remedial resources—they have accumulated a heterogenous mass of purging, sweating, heating, cooling, &c. medicines, and have told us, that such a remedy was good for such a complaint, and such another was excellent for the cure of some other disease, with but partial reference to the indications to be fulfilled by these various means. We mean the great leading indications, such as Sydenham meant when he said, that "the practice of physic chiefly consists in being able to discover the true curative indications, and not the medicines to answer them; and those that have overlooked this point, have taught empirics to imitate physicians." Again, says this sagacious physician, "whoever considers the matter thoroughly, will find, that the principal defect in the practice of physic, proceeds, not from a scarcity of medicines to answer particular intentions, but from the want of knowing the intentions to be answered. For an apothecary's apprentice can tell me, in a very short time, what medicines will purge, vomit, sweat, or cool; but a man must be much conversant in practice to be able to inform me, as certainly, which is the properest time of administering any particular remedy, in all the different stages of diseases, and throughout the course of the cure."

We will, with the reader's indulgence, expatiate a little on some defective points related to this subject, to be found in our writers on materia medica.

There has been a general concession on the part of all the late writers on materia medica, that our list of remedial substances, though retrenched of many inert articles which formerly encumbered it, is still too large—and that a further retrenchment is demanded. Availing ourselves of this very general judgment on the necessity of reform and retrenchment, our most assiduous efforts should be directed to this point. That there is ample room for such a process of extirpation, we think evident, from a very superficial glance at our catalogue of medicinal articles. Thus, in the edition of 1835, of Prof. Thompson's Elements of Materia Medica, we have upwards of four hundred and fifty separate articles noticed, as possessing sufficient medical virtues to be collocated as appropriate instruments of remedial efficacy. To be more explicit, Dr. Thompson, under the head of emetics, enumerates sixteen articles; under the same head, Dr. Chapman has eleven; Dr. Eberle sixteen; Barbier has a few less than either of the other high authorities. Under the head of cathartics, the first named author has fifty-four articles; the second named author, under the same head, places twenty-five distinct substances; Dr. Eberle puts twenty-four separate remedies down as cathartics; whilst Barbier swells the list to more than thirty. Now, it is very clear that more than one half of the articles enumerated by these authors as emetics, might be reformed out of the way, without the least detriment to the cause of enlightened therapeutics. Let us see if this remark cannot be made apparent to the reader. Take Dr. Chapman's list of articles possessed of emetic powers—they are as follows: 1. Callicocca Ipecacuanha. 2. Spirœa Trifoliata. 3. Euphorbia Ipecacuanha. 4. Sanguinaria Canadensis. 5. Nicotiana Tabacum. 6. Lobelia Inflata. 7. Scilla Maritima. 8. Tartris Antimonii. 9. Sulphas Cupri. 10. Sub-Sulphas Hydrargyri Flavus. 11. Zinci Sulphas. Of these we may, with great benefit to the art of curing the sick, take away more than two-thirds from our list of emetic medicines. Ipecacuanha, Tart. Antimon. Sulphate of Zinc—these we retain, and reject the others, as not adapted to fulfil, safely and with appropriateness of effect, our indications of cure, in cases requiring this class of remedial articles. The same retrenchment should be made to redeem the class of cathartics from the superfluous materials accumulated under that head.

The criticism, already passed on two classes of medicinal substances, will hold true of all the others. Is it not obvious from these facts, that a more precise and scientific simplification should obtain in our discussions on the materia medica? Besides, there is a very remarkable omission on the part of our American writers on this department of medical research, in reference to one of our most effective weapons of medical warfare. Is it not a singular pretermission on the part of these authors, that blood-letting is never brought forward at all, in a distinct and special manner, as a remedy in disease? Again, there is a very great oversight in their total silence respecting alterants, as a separate class of curative agents.—We know not a more signally triumphant resource of medical art than that which is found in the alterant power, exerted by several of our most potent remedies. Especially will this observation hold true in many chronic diseases. We are aware that those articles which pass under the designation of alterants, are treated of by both our American writers on materia medica, under other heads. But with most obvious impropriety, it will appear, we think, to the reflecting reader. Take mercury for illustration. Dr. Chapman, with an expressed hesitation, collocates mercury under the class of sialogogues. Dr. Eberle likewise places this great alterant under the same class—where it stands in solitary abstraction. Would it not have been far more philosophical, as comporting best with the acknowledged powers of the remedy, to have established a class to which it properly belongs, and to which other articles of the materia medica legitimately belong?

The salivant effect of mercury is but an incidental accompaniment to its alterant influence on the system, and it is therefore most unscientific to regard the remedy in an aspect so limited. It is a familiar truth to every practical physician, that the curative constitutional agency of the mineral is often secured, with little or no appreciable action exerted on the salivary glands.

The student of medicine, should he read any modern practical works on medicine or surgery, or attend lectures on these departments, will frequently read, or hear, allusions made to alterants, and he will be exceedingly perplexed to know what is meant by this class of medicines—how they act, what they are, and to what cases they are most applicable. And where shall he look for adequate information on this point? Why, he may find several pages devoted to this subject in the French Dictionary of Practical Medicine and Surgery—and a short article on alterants in the English Cyclopædia of Practical Medicine, and a shorter one in the American Cyclopedia of Practical Medicine and Surgery. If he is not satisfied with these rather meagre discussions of the question, then to have his mind well enlightened on this important matter, he must range up and down in his investigations, along the entire shelves of a well selected medical library of recent works on medicine and surgery.

Let the reader just examine the classification of medicinal substances, as laid down by most of our recent writers on the materia medica. What a sad dereliction, from that precise and perspicuous collocation, or distribution, which should obtain, is exhibited in those tables of classification, where remedies are arranged under the respective heads of vital agents, chemical agents, and mechanical agents. And the truisms that medicines will not act upon a dead body, nor in contradiction to the laws governing the living animal economy, are, with entire confidence of their value, every day pronounced by those who contemplate medicinal bodies as effecting the system in a chemical and mechanical manner. All medicinal powers act upon the vital endowments of the body, and induce changes only by their capability of modifying the organism through its essential aptitudes, or susceptibilities, of being impressed by foreign agents.

Professor Thompson, of the London University, now University College, and Professor Dunglison of the Jefferson Medical College, have both fallen into this mistake—and from this cause, they have made a most confused collocation of our remedial articles. Thus, Professor Thompson commences with vital agents, influencing the body generally, by operating on the nervous system—under this division are placed sedatives, refrigerants, narcotics, and antispasmodics. He, in the next place, treats of agents which act on the muscular and sanguiferous system, which are, tonics and astringents. In the third place, he discusses vital agents, which act on the secerning system, such as errhines, sialogogues, expectorants, emetics, cathartics, &c.

His chemical agents are escarotics, antacids, antalkalies, and antilithics.

The mechanical agents are demulcents and diluents.

All this appears to our minds very much like the old scholastic subtlety—a curious piece of mosaic refinement, entirely at variance with that axiom already announced, that all medicines act upon the powers of life, and not in virtue of any chemical law. The laws of physics cannot reign over the movements of life.

The following table of classification, exhibits the arrangement which we prefer.

I. Alterants.

*a* Anti-inflammatory.

*b* Anti-chacectic—or invigorating.

II. Evacuants.

*a* Blood-letting.
*b* Emetics.
*c* Cathartics.
*d* Diuretics.
*e* Antilithics.
*f* Emmenagogues.
*g* Expectorants.
*h* Anthelmintics.
*i* Diaphoretics.

III. Incitants, or Excitants.

*a* Stimulants.
*b* Narcotics.
*c* Antispasmodics.
*d* Tonics.
*e* Astringents.

IV. Derivatives; Revulsives; Counter-Irritants.

*a* Baths at various temperature.
*b* Frictions.
*c* Rubefacients.
*d* Epispastics.
*e* Suppuratives.
*f* Cauterizing counter-irritants.

Under the respective heads of the above table, all our medicinal substances can be arranged.

Our author has included alterants and blood-letting in his catalogue of remedial powers. We highly approve of this peculiarity about his work. Dr. Thompson has some very judicious remarks on blood-letting, but is silent concerning alterants. We recommend to the reader's consideration, the suggestions of our author on both of these topics. We regret that he did not say more on both of these deeply interesting practical points.

In his concluding observations, our author has the following judicious reflections.

"In all cases of disease, the physician must investigate the nature of the pathological action going on in the tissues.—This, as I have elsewhere remarked—must be the point of departure for his therapeutical indications; and a wise knowledge of the properties of his medicinal agents will enable him to carry into effect those indications; but let him not vacillate. Let his therapeutics be guided by great general principles; and let him not fly from one indication to another, because the pathological condition may not be, or may not seem to be, yielding. If his principles be accurate, his indications will correspond; and if he be not satisfied with his indications, there will be but little safety for the patient, in the empiricism that must necessarily be the rule of conduct under such circumstances. It is an unfavorable index of the attainment of a physician, when he places undue store on some formula, or agent, for the cure of particular pathological conditions. It is *prima facie* evidence of faulty observation, and of defective induction. It indicates a mind that searches after facts—too often 'false'—rather than after great principles; that is swayed by prepossessions, rather than by true experience, and that ought not to be trusted on trying or unusual occasions. To the possessor of such a mind, the advice to a young author—if he had written any thing which he thought particularly fine, to strike it out—might not be inappropriate. The loss of the result of the practitioner's observation, true or false, would be but trifling, whilst the reception of a false fact, and its admission as ground of action, might be most unfortunate.

"The present is a period, eminently characterized for the accumulation of clinical observations, and much valuable information has been collected: yet, it is to be feared, the advantages, in this way obtained, have not been without countervailing results; and there are, perhaps, too many evidences of the minds of observers having been narrowed down to simple observation of occurrences, instead of being devoted to great general views. Facts have occupied the mind in the place of induction. The senses have been engaged, whilst the higher powers of the intellect have, too often, been permitted to remain dormant."

"Facts are the foundation of all true theory—of all great principles of therapeutics—but such facts must be indubitable. The science of medicine has suffered greatly from the questionable and the false."

"Where the pathological lesion is understood, the therapeutical indication is clear; and, in general, easily fulfilled. No necessity will exist for complex combinations of different classes of medicinal agents; or of different agents of the same class."

"It has been well remarked, 'that a medicinal prescription should carry upon its face an air of energy and decision, and speak intelligibly the indication which it is to fulfil—and that it may be laid down as a position, not in much danger of being controverted, that where the intention of a medical compound is obscure, its operation will be imbecile.' There is, doubtless, truth, too, in the opinion entertained by some, that a practitioner's professional abilities may be estimated somewhat by the character of his prescription—the scientific physician not permitting any agent to enter, without some satisfactory reason for its admission. Where no such reason can be adduced, there is usually some deficiency on the part of the practitioner."

"Where complexity is indulged, it is obviously impracticable to test, by any kind of experience, the properties of our therapeutical agents, or to discriminate the main sanative ingredient, from such as are of no efficacy, or may even counteract it."

"Connected with this subject, Professor Bigelow, of Boston, has some judicious remarks, which strikingly exhibit the fallacious mode of reasoning, adopted by too many, in their investigation of disease, and of the proper adaptation of remedies."

'The foundation of all knowledge is truth. For truth, then, we must earnestly seek, even when its developements do not flatter our professional pride, nor attest the infallibility of our art. To discover truth in science is often extremely difficult; in no science is it more difficult than in medicine. Independently of the common defects of medical evidence, our self-interest, our self-esteem, and sometimes even our feelings of humanity, may be arrayed against the truth.'

'Nothing can be more illogical, than to draw our general conclusions, as we are sometimes too apt to do, from the results of insulated and remarkable cases; for such cases may be found in support of any extravagance in medicine; and if there is any point in which the vulgar differ from the judicious part of the profession, it is in drawing premature and sweeping conclusions, from scanty premises of this kind. A great deal of our practical literature consists in reports of interesting, extraordinary, and successful results, published by men who have a doctrine to establish, or a reputation to build. Few authors, says Andral, have published all the cases they have observed, and the greater part have only taken the trouble to present to us those facts, which favor their own views. A prevailing error among writers on therapeutics, proceeds from their professional, or personal, reluctance to admit that the healing art, as practised by them, is not, or may not be, all sufficient, in all cases: so that on this subject they suffer themselves, as well as their readers, to be deceived. Hence, we have no disease, however intractable or fatal, for which the press has not poured forth its asserted remedies. It is only when, in connexion with these flattering exhibitions, we have a full and faithful report of the failures of medical practice, in similar, and in common cases, setting forth, not only the truth, but the whole truth, that we have a basis sufficiently broad to erect a superstructure in therapeutics, on which dependence may be placed.'

The above extract is taken from 'A Discourse on Self-Limited Diseases,' delivered before the Massachusetts Medical Society, at the annual meeting, May 27, 1835, by the distinguished Professor Bigelow.

We feel that it is time to bring our review of Professor Dunglison's work to a close. In the spirit of a free, candid, and fearless criticism, we have commented on, what we considered to be, its inherent defects, glaring redundancies, and evident derelictions from the genius of a sound philosophy in Medical Science. We may have erred in the judgment passed upon the work. Our adjudication, if at all erroneous, has not been perverted, however, by any unkind prejudices, or darkening personal feelings. We entertain a warm partiality for the author; though not from any considerations other than those of a professional kind, for we have not the happiness of numbering him among our personal acquaintances. For his great merits as a scholar, lecturer, and author, we feel the profoundest respect, and for his contributions to medical literature, he deserves, and will receive, the grateful benedictions of thousands in our country. May his days be prolonged on earth, and may he never compromise his well merited reputation by any future work, which shall betray equal marks of hasty composition, of crude arrangement, and inelegant style, as does his "General Therapeutics." J. P. H.

